# Intelligence

**Published:** 1830-01

**Authors:** 


					INTELLIGENCE.
MONTHLY REPORT OF DISEASES.
During the course of the last month, the general character of disease has
been such as might be expected from the severity of the weather. Rheu-
matism, catarrhs, and inflammation of the lungs, have been frequent. In two
cases of asthma, we have certainly derived advantage from the exhibition ot
the tincture of the Lobelia inflata, which has recently been so much extolled
by the American physicians. Two cases of that affection in young children,
which has, we think, very improperly been called "cerebral croup," have
also fallen under our notice. In both there were the croupy breathing, the
thumbs firmly turned in upon the palms of the hand, and the inverted state
of the feet. Purgatives and strict attention to diet quickly relieved these
symptoms.
METEOROLOGICAL JOURNAL,
By Messrs. Harris and Co. Mathematical Instrument Makers, 50, High Holboru.
20
21
22
23
24
25
26
27
28
29
30
Dec.
1
2
3
4
5
6
7
}? o
11
Thermom.
Barometer.
to ^
30.22
.15
29.84
.47
.53
.42
.73
.63
.63
.68
.71
.71
.64
.68
.67
30.14
.37
.24
.18
.24
.04
.04
.05
.08
.16
.22
.15
29.85
.50
.73
30.20
.05
29.58
.57
.44
.58
.75
.63
.68
.70
.71
.68
.61
.71
.82
30.27
.29
.15
.24
.14
.00
.04
.07
.12
.19
.04
29.71
.63
.70
DeLuc'i
Hygrom
Winds.
S
N
N
S\V
N\V
ENE
.NE
E
E
NE
N W
SE
SE
E
ESE
SSE
SSE
SE
SE
S
N
NE
SSE
sw
sw
ssw
sw
NNE
NE
SE
NNE
N
W
SW
NNU'
NE
E
E
NE
N
NW
SE
SE
ESE
ESE
SSE
S
SE
SSE
W
NE
ENE
S
SSW
SW
S
WSW
NE
NE
ENE
NE
Atmosphe ric Variations,
9 a.m. 2 p.m. lOp.m
Fog
Sbow'ry
Fine
Cloudy
Foggy
Cloudy
Foggy
Foggy
Foggy
Fine
Foggy
Fine
Foggy
Th. Fog
Fog
Foggy
Fine
Th. Fog
Kain
Clondy
Cloudy
Shoiv'ry
Cloudy
Sleet
Cloudy
Cloudy
Fine
Foggy
Fine
Fine
Foggy
Fine
Fine
Fine
Th. Fog
Foggy
Fine
Fine
Fine
Fine
Th. og
Fin e
Show'ry
Shovv'ry
Kain
F.&Mist
Cloudy
Sleet
Fine
Fine
Shovv'ry
Foggy
Fine
Foggy
Fine
Fine
Fine
Fine
Fine
Foggy
Fine
Fine
Foggy
Fine
Fiue
The gauge having frozen, the quantity of Rain cannot be given.

				

